# Hyperosmotic stress induces cell-dependent aggregation of α-synuclein

**DOI:** 10.1038/s41598-018-38296-7

**Published:** 2019-02-19

**Authors:** Alexandra M. C. Fragniere, Simon R. W. Stott, Shaline V. Fazal, Maria Andreasen, Kirsten Scott, Roger A. Barker

**Affiliations:** 1 John van Geest Centre for Brain Repair, E.D. Adrian Building, Forvie Site, Robinson Way, Cambridge, United Kingdom; 2 Department of Chemistry, University of Cambridge, Lensfield Road, Cambridge, CB2 1EW United Kingdom; 3 Interdisciplinary Nanoscience Center (iNANO), Aarhus University, Gustav Wieds Vej 14, 8000 Aarhus, Denmark; 4 Wellcome Trust-MRC Stem Cell Institute, Cambridge, United Kingdom

## Abstract

The aggregation of alpha-synuclein (α-syn) is a pathological feature of a number of neurodegenerative conditions, including Parkinson’s disease. Genetic mutations, abnormal protein synthesis, environmental stress, and aging have all been implicated as causative factors in this process. The importance of water in the polymerisation of monomers, however, has largely been overlooked. In the present study, we highlight the role of hyperosmotic stress in inducing human α-syn to aggregate in cells *in vitro*, through rapid treatment of the cells with three different osmolytes: sugar, salt and alcohol. This effect is cell-dependent and not due to direct protein-osmolyte interaction, and is specific for α-syn when compared to other neurodegeneration-related proteins, such as Tau or Huntingtin. This new property of α-syn not only highlights a unique aspect of its behaviour which may have some relevance for disease states, but may also be useful as a screening test for compounds to inhibit the aggregation of α-syn *in vitro*.

## Introduction

The processes underlying protein aggregation are a major focus of neurodegenerative research. Amyloid fibrils, the most stable forms of ordered protein aggregates, are features of a number of common neurodegenerative disorders such as Alzheimer’s, Parkinson’s (PD) and Huntington’s disease^1^. These amyloid fibrils are formed by intrinsically disordered proteins (IDP)^2^, which possess little or no 3D structure in their native state and one such example is alpha-synuclein (α-syn) which forms amyloid fibrils that contribute to the cytoplasmic inclusions, that represent the pathological hallmarks of α-synucleinopathies, such as PD, dementia with Lewy bodies, and multiple system atrophy^3^.

α-syn is an IDP^4^ expressed in most tissues, but it is particularly enriched in the brain where it localises primarily in presynaptic terminals^5^. The physiological function of α-syn still remains poorly understood, although it does appear to have a critical role in membrane interactions^6^. α-syn is composed of 140 amino acids which can be divided into three domains: (1) an N-terminal region containing six imperfect KTKEGV repeat motifs^7^ which form amphipathic helices upon membrane binding^8^, (2) a hydrophobic central region prone to form ß-sheets^9^, and (3) a C-terminal acidic region rich in prolines and involved in protein-protein interactions. Upon aggregation, a large fraction of α-syn undergoes a transition from a random-coil structure to the cross-β conformation typical of amyloid fibrils^10^. Although the precise 3D structure of α-syn fibrils remains unknown, they can be divided into two structurally distinct regions: a central rigid core rich in β-sheets that are aligned in parallel along the fibril axis, and a superficial flexible coat composed of the N- and C-terminus of α-syn^11^. Despite these important advances in our understanding of α-syn fibril structures, the cellular mechanisms underlying their formation remain elusive.

It has long been appreciated that water plays a key role in protein folding, stability and activity^12^. However, the actual effect of water on protein aggregation has not been comprehensively investigated in cells, with most of the evidence originating from molecular dynamic simulation studies^13–16^. In these studies, the first stage of fibril formation involves water molecules being expelled from the vicinity of the monomeric protein rich in hydrophobic residues^14^. This led Chong and Ham^13^ to suggest that the extent to which a protein is soluble or prone to aggregation relies intrinsically on its affinity for water in its monomeric state. This water expulsion step may precede, and in effect prompt, the subsequent assembly of monomeric proteins into fibrils.

The primary objective of the current study was to test these simulation results and experimentally address whether water expulsion can initiate protein aggregation in cells. We expressed human α-syn in neuronal cells and induced a rapid efflux of intracellular water by applying an acute osmotic gradient across the plasma membrane. This treatment led to the rapid formation of α-syn aggregates which were not seen when the plasma membrane was permeabilised using detergent. Similarly, when each osmolyte was added directly to purified α-syn outside of a cellular environment, aggregation did not occur. These results provide experimental evidence that hyperosmotic stress can induce aggregation of α-syn in cells.

## Results

### Hyperosmotic stress induces aggregation of α-syn in cells

**Figure 1:**
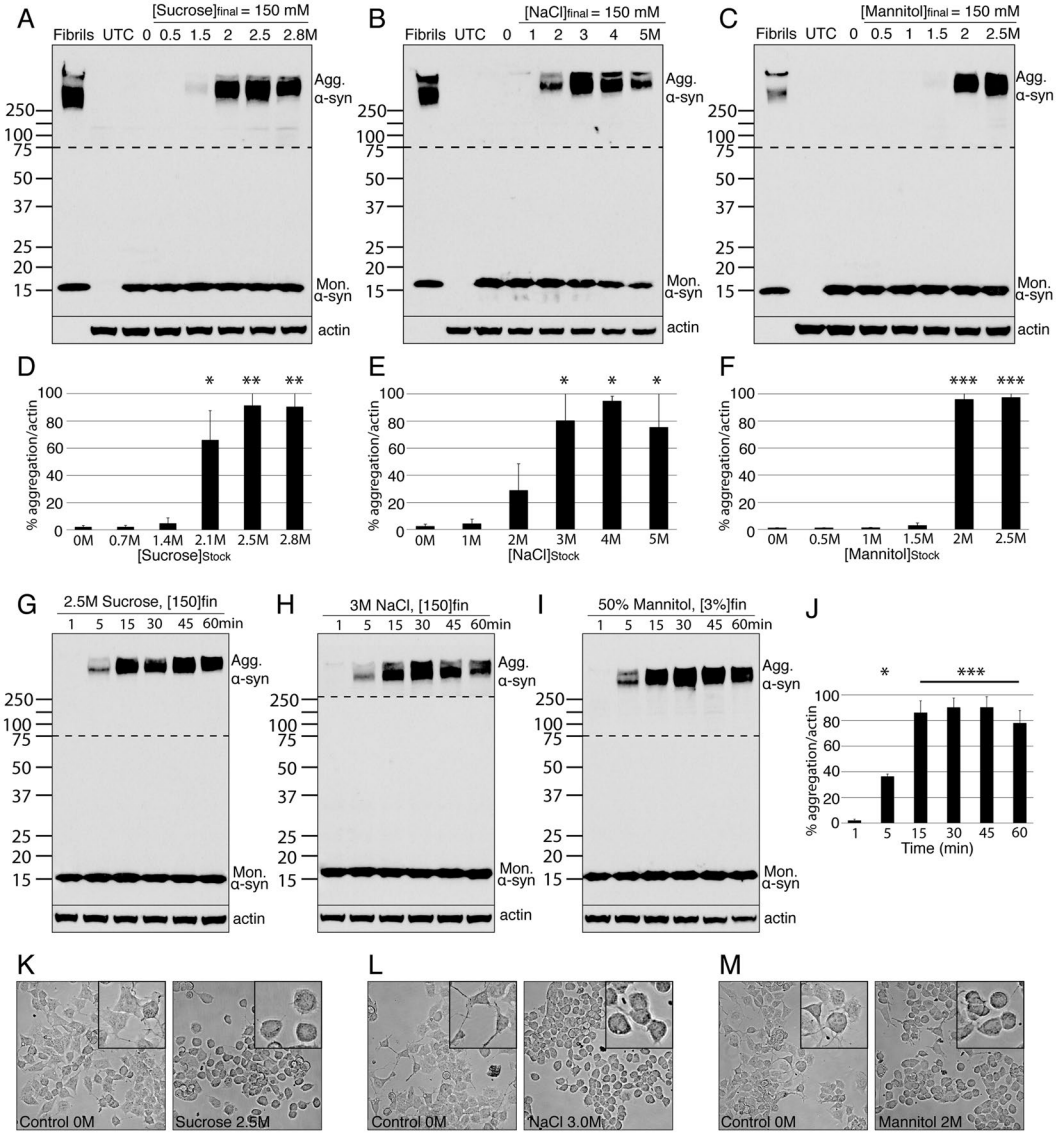
Hyperosmotic stress induces aggregation of α-syn. (**A**–**C**) Western blot gels illustrating the aggregated alpha synuclein (Agg. α-syn) and monomeric alpha synuclein (Mon. α-syn) from cell lysates collected after each treatment. In the first lane, 5pmoles of α-syn fibrils were used as a positive control. In the second lane, whole cell extract from cells overexpressing GFP and treated with the highest concentration of osmolytes were used as a negative control (UTC). (**D**–**F**) Bar graphs of the quantifications from the corresponding gel directly above. Error bars represent standard errors between results obtained with N2a and HEK cells. (**G**–**I**) Western blot gels presenting the time course of Agg. α-syn over time after each treatment. (**J**) Bar graph presenting the quantifications of the average of the sucrose, NaCl and Mannitol time course experiments (in panel G–I). (**K–M**) Light field microscopy images of cells *in vitro* following the indicated treatment. Insets of high magnification images are provided to demonstrate the morphology of the individual cells. *p < 0.05, **p < 0.005, ***p < 0.0005. The panel size in K-M represents 100 × 100 µm, 25 × 25 µm in the insets. The portion of the blots above the dashed lines was exposed for a longer time compared to the part of the blot below the dashed line (Fig. S4).

To assess the capacity of water expulsion to induce aggregation of human α-syn, we applied three different osmolytes to N2a cells overexpressing the protein: a sugar (sucrose), a salt (NaCl), and an alcohol (mannitol). A range of stock concentrations was prepared for each osmolyte, according to their solubility in water. The stock solutions were added directly onto the cells (drop by drop; Fig. S1), to a final concentration in the supernatant of 150 mM (Table 1). This protocol allowed us to achieve a fast and transient water efflux from the cells: as the drop diluted into the supernatant, the osmolyte equilibrated to a low concentration, and with this the osmotic pressure dropped. Changes in cell morphology illustrated the transient change in the osmotic pressure - from elongated to a more rounded shape immediately after treatment (Fig. 1K–M) - back to elongated after dilution of the osmolyte in the extracellular medium. After the osmotic shock was applied, the whole cell extract was collected and analysed by western blot (WB). Recombinant α-syn fibrils were used as a positive control for the expected pattern of α-syn aggregates on WB. Cells overexpressing GFP, instead of α-syn, and treated with the highest concentration of osmolytes were used as a negative control (UTC). α-syn aggregation was detected after treatment with each osmolyte (Figs 1A–F and S6; Sucrose ANOVA P = 0.0013, NaCl ANOVA P = 0.016, Mannitol ANOVA P < 0.0005). Attempts to visualise this aggregation using immunohistochemical staining failed due to high background staining in control cells, and so we focused our experiments on WB analysis. This effect was not cell line specific as we found the same effect for transfected HEK cells (Figs S2 and S6). The aggregates formed by the cells were highly stable, exhibiting SDS and Urea resistance (Fig. S3), two properties characteristic of α-syn fibrils. Aggregation was obtained when a stock solution of at least 2 M was used for all three osmolytes (Fig. 1D–F). Furthermore, time-course studies showed that the aggregates formed at the same speed when cells were exposed to sugar, salt or alcohol, and were detected within 5 minutes of the hyperosmotic shock (Fig. 1G–I), peaking at 15 minutes (Fig. 1J; ANOVA P < 0.0005). Taken together, these results suggest that osmotic shock - regardless of the agent driving it – causes the aggregation of α-syn through drawing water molecules out from the cells.

**Table 1:** A table presenting the amount of osmolyte added onto the supernatant of cells (2 mL) for every concentration of stock solution.

**Sucrose**					
Stock solution [M]	0.5	1.5	2	2.5	2.8
Amount added onto cells [μL]	600	200	150	120	107
**NaCl**					
Stock solution [M]	1	2	3	4	5
Amount added onto cells [μL]	300	150	100	75	60
**Mannitol**					
Stock solution [M]	0.5	1	1.5	2	2.5
Amount added onto cells [μL]	600	300	200	150	120

### Hyperosmotic stress induced aggregation is specific for α-syn protein

**Figure 2:**
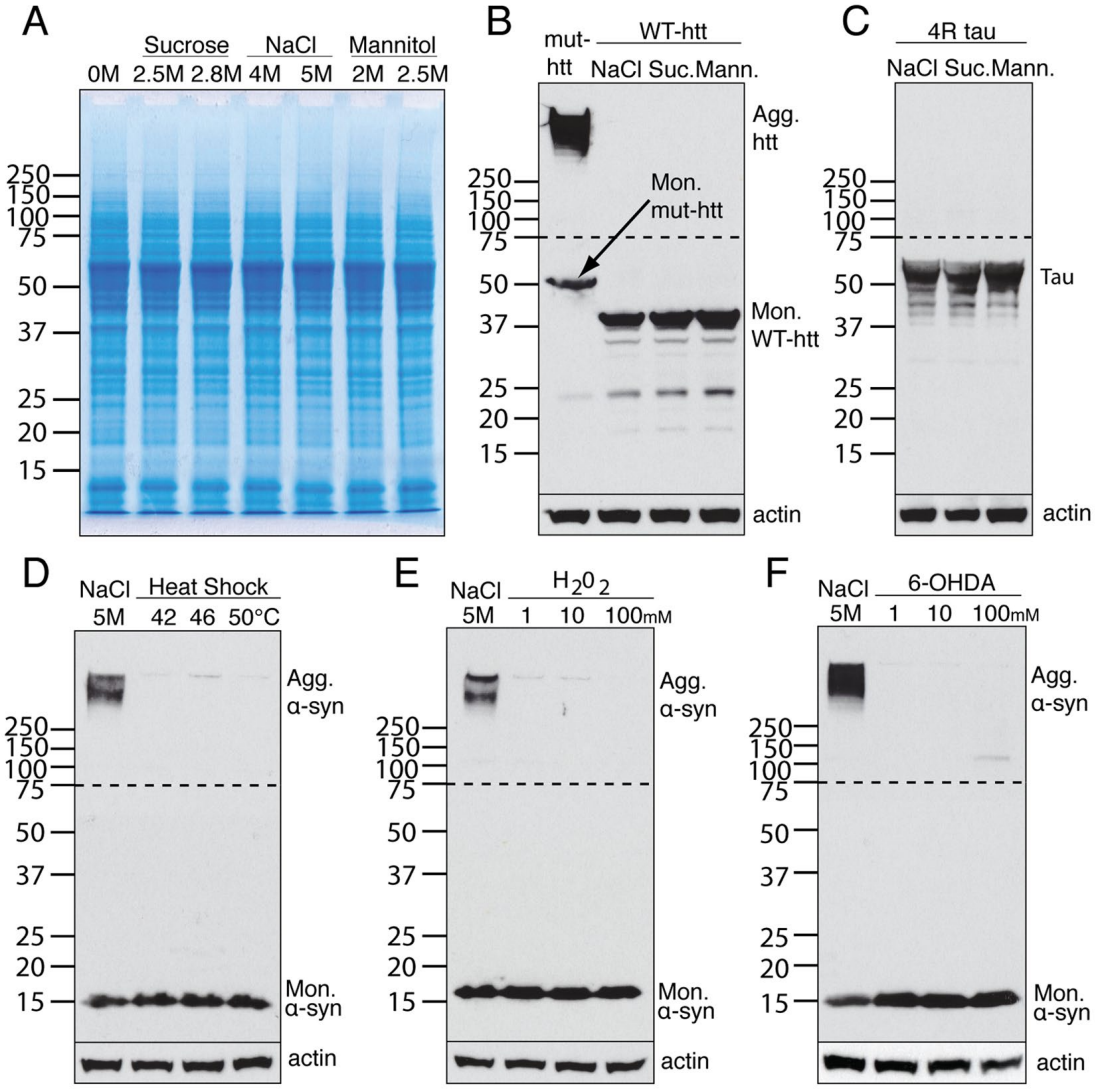
The effect is specific to α-syn and to hyperosmotic stress. (**A**) The complete range of endogenous cellular proteins analysed by Coomassie gel following osmotic shock from sucrose, NaCl or mannitol. (**B**,**C**) Western blot analysis of Huntingtin (htt)  and Tau protein following osmotic shock from NaCl, sucrose (Suc.) or mannitol (Mann.). **(D**–**F**) Western blot analysis of α-syn aggregation following different levels of heat shock, hydrogen peroxide (H_2_O_2_) or 6-hydroxydopamine (6-OHDA). The portion of the blots above the dashed lines was exposed for a longer time compared to the part of the blot below the dashed line.

To determine whether the solubility of other neurodegenerative disease-associated cellular proteins was similarly affected by hyperosmotic stress in this system, we looked at a complete range of endogenous cellular proteins by Coomassie gel analysis. The whole cell extract showed no obvious differences between untreated and treated cells at this level of detection (Fig. 2A). Next, we analysed the effect of osmotic shock on two other aggregation-prone proteins, Tau and Huntingtin (htt), when overexpressed in the same cells. Hyperosmotic stress failed to induce aggregation of either Tau or htt. (Fig. 2B,C). Interestingly, hyperosmotic stress also had no effect on the solubility of GFP-tagged human α-syn (data not shown). These results suggest that the effect of hyperosmotic stress on protein aggregation is specific to untagged α-syn.

To assess whether the ability to induce α-syn aggregation was specific to hyperosmotic stress, α-syn overexpressing cells were subjected to three other types of stress: heat shock, oxidative stress, and a neurotoxin that is used to create models of PD, 6-OHDA. α-syn remained monomeric when cells were heated up to 50 °C (Fig. 2D), exposed to high concentration of H_2_O_2_ (Fig. 2E), or treated with toxic levels of 6-OHDA (Fig. 2F). These results confirmed that α-syn does not spontaneously aggregate in cells, even when overexpressed, and remains soluble when the cells are under different types of stress, but appears to be specifically vulnerable to hyperosmotic stress.

### The hyperosmotic stress induced aggregation of α-syn is cell-dependent

**Figure 3:**
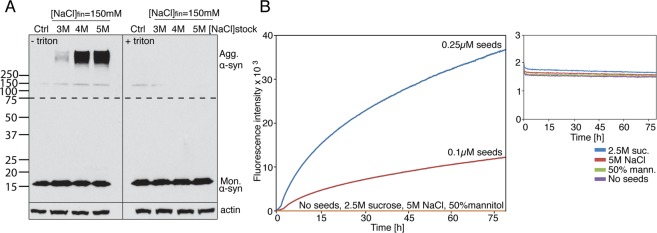
α-syn aggregates form in a cell-dependent manner. (**A**) Western blot analysis of α-syn overexpressing cells, treated with and without triton before different concentrations of NaCl induced osmotic shock. (**B**) Thioflavin T (ThT) fluorescence analysis of 50 µM recombinant α-syn treated with drops of 2.5 M sucrose, 5 M NaCl or 2.5 M mannitol to a final concentration of 150 mM. Seeds made from recombinant α-syn were used as positive controls. Inset shows magnification of the flat ThT readings following treatment of recombinant α-syn with sucrose, NaCl or mannitol. The portion of the blots above the dashed lines was exposed for a longer time compared to the part of the blot below the dashed line.

To confirm that the observed aggregation was a result of the cellular response to the hyperosmotic shock, and not due to direct protein-osmolyte interaction, we used detergent to disrupt the cell membrane and therefore prevent the osmotic response. α-syn overexpressing cells were collected in a high-density suspension culture inside eppendorf tubes. Aggregation was induced by adding one drop of NaCl into the cell solution to a final concentration of 150 mM. However, when triton was added to the cell solution before the osmotic shock, α-syn remained soluble (Fig. 3A). To exclude the possibility that the aggregation was suppressed because of the dilution of the protein into the extracellular medium after membrane permeabilisation, the same experiment was repeated using recombinant α-syn at 50 μM, a concentration much higher than that which can be achieved by overexpression in mammalian cells. The results were analysed using Thioflavin T **(**ThT) fluorescence, a method commonly used to monitor aggregation of recombinant α-syn. All three osmolytes failed to induce aggregation of recombinant α-syn (Fig. 3B). Collectively, these results highlight the importance of the cellular response to the change in osmotic pressure in driving α-syn aggregation, and rules out any direct protein-osmolyte interaction.

### Osmotic shock induced α-syn aggregation does not cause cell death

**Figure 4:**
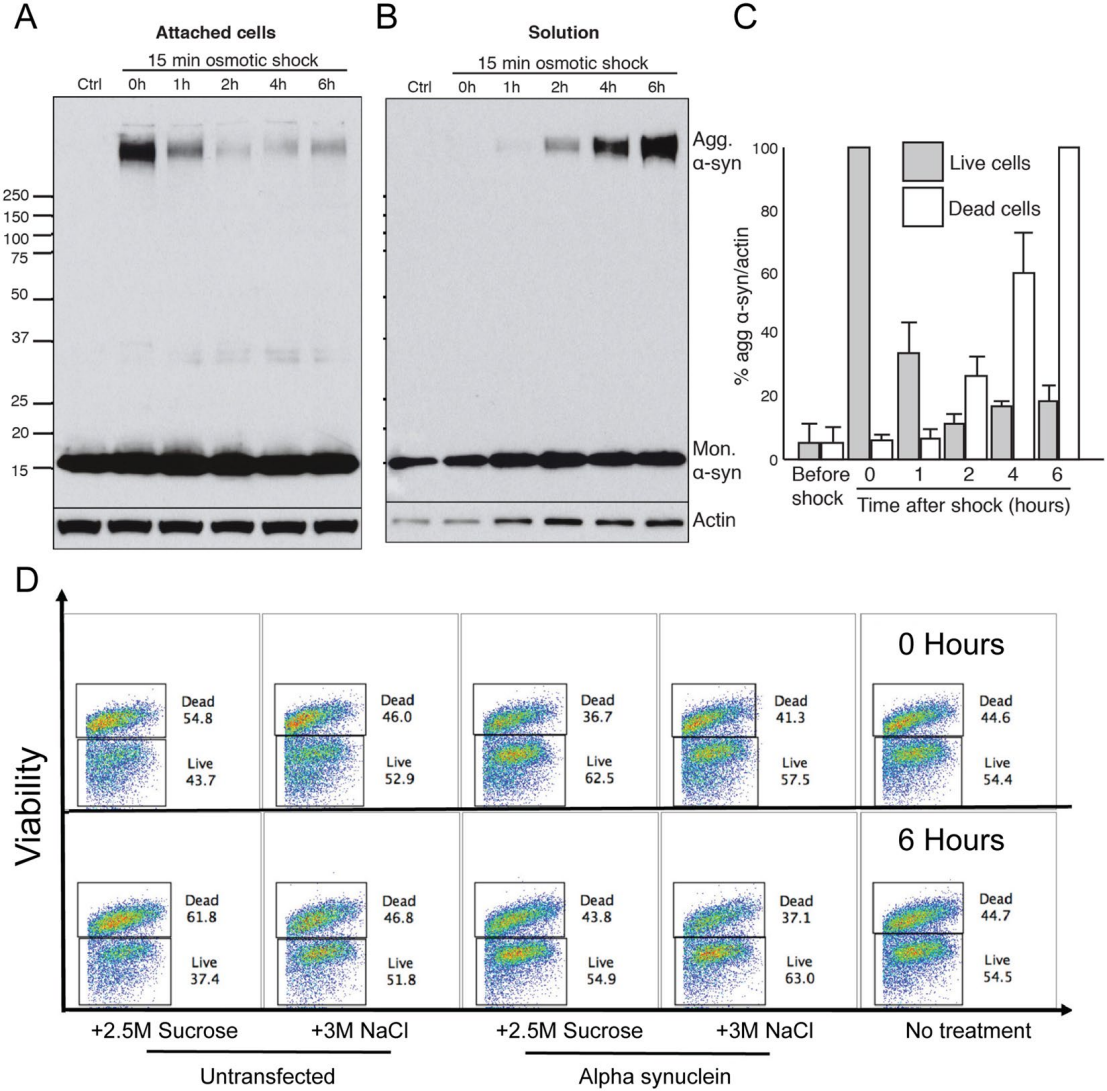
α-syn aggregation does not induce cell death. (**A**) Western blot analysis of live cell lysates collected at different time points following osmotic shock. (**B**) Western blot analysis of dead cell lysates collected at different time points following osmotic shock. (**C**) Bar graph demonstrating the percentage of α-syn aggregation/actin, with data presented from the live cells and dead cells analysis (panel A & B, respectively). (**D**) An example of the FACS plots derived from cell death experiments.

To analyse cell fate following aggregate formation, cells were collected at various time points following a 15-minute osmotic shock with sucrose. Drops of 2.5 M sucrose were added onto cells to a final concentration in the extracellular medium of 150 mM. The extracellular medium was changed after the 15-minute shock to remove the added sugar. The medium was not changed for the 0 h time point. For each time point, cell extract from both living cells, attached to the bottom of the dish, and dead cells, floating in the supernatant, were collected. Analysis of the aggregated α-syn signal revealed that, within 2 hours of the osmotic shock, most of the aggregated protein had disappeared from the cell extract of living cells (Fig. 4A,C), and appeared in the cell extract of dead cells (Fig. 4B,C). However, no change was observed for monomeric α-syn (Fig. 4A and B). These results indicated that the population of dead cells was highly enriched in cells containing α-syn aggregates, and suggested that aggregate formation was toxic to cells.

To determine whether hyperosmotic stress induced aggregation of α-syn was actually causing cell death, we conduced a series of experiments to investigate this, including FACS analysis (Fig. 4D), LDH assay (Fig. S5B) and propidium iodide staining (data not shown). All of these methods failed to demonstrate any difference in cell death between the conditions tested (untransfected cells with no treatment, untransfected cells treated with osmolyte, and α-syn transfected cells treated with osmolyte). Collectively the findings show that osmotic shock induced α-syn aggregation does not cause significant cell death within the total cell population.

### Failure to detect α-syn aggregation *in vivo*

**Figure 5:**
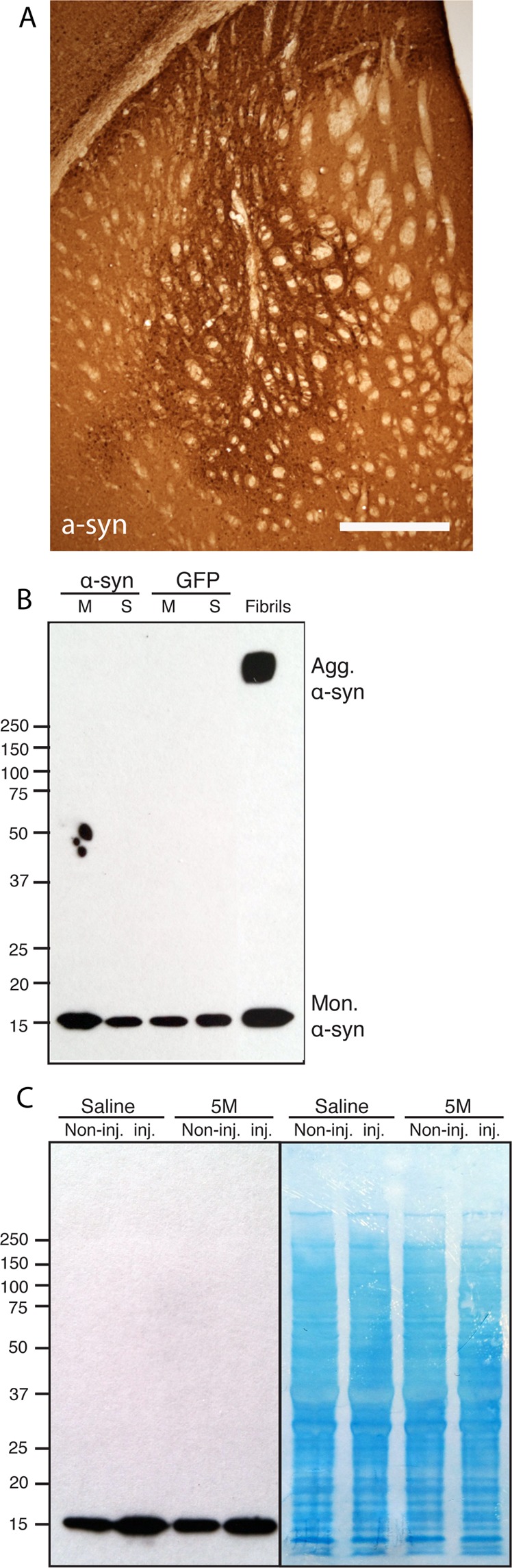
α-syn aggregation not detected *in vivo*. (**A**) Immunohistochemical staining of virally over-expressed α-syn in the striatum 8 weeks post surgery. (**B**) Western blot analysis of α-syn aggregation in the midbrain (M) and striatum (S) of AAV-α-syn and AAV-GFP injected mice. (**C**) Western blot analysis of α-syn aggregation on the injected striatum and noninjected stiatum from saline and 5 M NaCl injected animals. Coomassie gel analysis demonstrated no difference. Scale bar in A represents 350 µm.

To determine if this hyperosmotic stress induced aggregation could be observed *in vivo*, we delivered 5 M NaCl into the striatum of WT mice that were injected unilaterally in the striatum with a Adeno-associated virus (AAV) encoding human-α-syn 8 weeks earlier (Fig. 5A). A control group was injected with AAV-GFP. The mice were sacrificed at different time points after the NaCl delivery (5 minutes, 10 minutes and 30 minutes; n = 3 at each time point). Normal saline was used as a control on a group of AAV-α-syn injected mice (n = 3 at each time point). Their brains were removed and the striatum and ventral midbrain were dissected out for WB analysis. We detected no aggregation in any of the tissue samples at any of the different time points using this approach (Fig. 5B,C). Whole cell extract showed no obvious differences between the groups either (Fig. 5C).

Assuming that the spread of 5 M NaCl was not sufficient or robust enough to induce an effect, we next took the brains of mice overexpressing human α-syn under the control of the thy1 promoter (mThy1-hSNCA, line 15; Jackson labs) and placed 500 μm thick coronal slices directly in 5 M NaCl solution. The slices were extracted 1, 5 or 10 minutes later, and prepared for WB analysis. Again, we detected no aggregation at any time points using either technique (data not shown). These results suggest that the hyperosmotic stress induced aggregation of α-syn does not occur *in vivo* in the same rapid manner as that observed in cells *in vitro*.

## Discussion

‘If you make monomers wet they don’t turn into polymers’ - While popular literature has been quick to point out the importance of hydration in polymerisation of monomers^16^, experimental studies have left the effect of the surrounding water largely unexplored when trying to understand the cellular mechanism of protein aggregation. Indeed, most experiments have been interpreted solely from the perspective of proteins, ignoring the role of water as an active constituent of the cell^10,17,18^. In the present study, we have experimentally addressed this in cells with respect to α-syn aggregation.

We found that hyperosmotic stress caused by three different osmolytes (sugar, salt and alcohol) induced α-syn aggregation, in a cell-dependent manner. The effect was not due to direct protein-osmolyte interactions, and it was also specific to α-syn as other neurodegeneration-related proteins (Tau or htt) did not exhibit similar aggregation using this system. Interestingly, following the osmotic shock, the aggregated α-syn disappeared from the population of living cells and appeared in the population of dead cells, suggesting that aggregate formation was toxic to cells. Investigations of cell death, however, failed to demonstrate any clear difference between the conditions, indicating that osmotic shock and aggregation of α-syn did not appear to induce cell death. These results suggest that the shift in aggregates from live cells to dead cells that was observed in the initial experiment may simply be due to the excretion of aggregated α-syn by live cells.

Our inability to demonstrate hyperosmotic stress induced aggregation *in vivo* or *ex vivo* could be the result of several factors. First, the spread of the viral transfection, production and level of transgene expression, and volume of 5 M NaCl utilised in the *in vivo* study may not have been enough to cause or detect any effect. In addition, the *in vivo* extracellular environment is more strictly regulated (by astrocytes and glial cells) than the cell culture conditions. In order to study more rigorously the aggregation phenomenon within brain matter, we utilised a transgenic mouse that ubiquitously over-expresses human α-syn and submerged brain slices from those mice in 5 M NaCl *ex vivo*. However, this approach also failed to elicit aggregation, which leaves open the question as to whether hyperosmotic stress induced aggregation occurs *in vivo*.

The cell assay, however, represents a novel technique for exploring α-syn aggregation and potentially screening for new therapeutics targeting this process. By treating cells that ubiquitously overexpress human α-syn with compounds of interest and then inducing hyperosmotic stress, quantiative measures of aggregation could be made. Alternatively, whole-genome, random activation CRIPSR-based screening studies (in which one gene is mutated in each human α-syn expressing cell) could be conducted to identify genes that may reduce the aggregation process.

In studies using bacterially expressed and purified α-syn outside a cellular environment, osmolytes have generally been considered to function as protein stabilizers due to their ability to inhibit protein aggregation^19,20^. In these experiments, where the protein comes in direct contact with the osmolyte, it has been demonstrated that the manipulation of acidity and salts can significantly influence the rates of α-syn aggregation^21,22^. Exposure to high-salt buffers result in antiparallel intramolecular conformations of purified α-syn variants^23^.

In the present study, the protein is in a cellular environment and the osmolyte is used to elicit a cellular response and remove water molecules from around the protein. Investigations exploring the effects of water and dehydration on α-syn in cells have been very limited. A study on htt protein found that spontaneously occurring aggregation of mutant htt was exacerbated after cells were treated with 0.5 M sorbitol (for at least 48 hours even after returning the cells to iso-osmotic media)^24^. The effect was shown to be specific to mutant htt as WT htt, which is not aggregation-prone, was not affected by the treatment (similar to the results of our study). And more recently, *in vivo* experiments have been published demonstrating that high and low osmotic conditions can influence the aggregation of α-syn in a transgenic NL5901 strain of C. elegans^25^. Under those sustained conditions, however, autophagic activity was significantly reduced which may have resulted in the inefficient clearance of α-syn, leading to accumulating and aggregating.

While there are few experimental studies on water and aggregation, a number of molecular dynamic (MD) simulation studies provide in silico evidence on how water controls the assembly of aggregation-prone proteins into fibrils. MD simulations have been used to study the hydration patterns of prion protein (PrP) and found that the protein surface presented regions where the hydration layer was disrupted: rather than forming a stable shell around the protein surface, the water molecules were rapidly exchanged with the bulk water of the cell^26^. The behaviour of these loosely attached hydration waters appears to have been crucial for the aggregation of PrP into fibrils. Interestingly, Fernandez at al. found that hydration layer defects were prevalent in aggregation-prone proteins, including prion, α-syn, amyloid-beta, compared to non aggregation-prone proteins^27^. Given that these regions are dramatically stabilized by the removal of water, they are susceptible to water attack; upon water expulsion, sticky patches on the protein surface are created which promote protein aggregation. Simulations have also found that water expulsion from the surface of an amyloid peptide preceded, and in effect prompted, the subsequent hydrophobic collapse and fibril formation^11,28^. Finally, a simulation on 43 intrinsically disordered proteins and 15 natively folded proteins confirmed the crucial role of hydration in determining the aggregation propensity of proteins^12^. They found that the extent to which a protein remained soluble, or became aggregation-prone, relied intrinsically on its affinity towards water in its monomeric state. The hydration of a protein, rather than the protein itself, was the predominant determinant of the aggregation propensity.

These simulation studies provide a theoretical framework to understand how water displacement can induce protein aggregation. The release of hydration water molecules from around the surface of aggregation-prone protein is thermodynamically favourable and provides free energy to initiate protein aggregation. In our system, water displacement was achieved using osmotic pressure. We propose that the observed aggregation is a result of the efflux of loosely attached water molecules from the hydration layer, exposing sticky patches that are stabilised upon interaction with other proteins, leading to aggregate formation.

In summary, we have shown that hyperosmotic stress induces aggregation of α-syn *in vitro*, and we believe that this assay will be a useful tool in the screening for novel compounds that have anti-aggregation properties.

## Methods

### Animals

All experiments in mice were performed in accordance with the UK Animals (Scientific Procedures) Act 1986 and ARRIVE guidelines. Animal licences were approved by the Home Office and the University of Cambridge’s Animal Welfare & Ethical Review Body Standing Committee. Experiments were performed under Home Office licences PPL 80/2366 and 70/8411.

### Cell Culture, Constructs and Transfections

Neuro-2a (N2a) cells and Human embryonic kidney cells 293 (HEK) were cultured in DMEM (Gibco) supplemented with penicillin, streptomycin, and 10% fetal bovine serum (FBS). Full-length WT human α-synuclein (α-syn) was generated by PCR and cloned into pCMV-myc (Clontech). The myc tag of the pCMV-myc vector was deleted to create the untagged αsyn construct. The construct was verified by sequencing. The WT and mutant exon 1 huntingtin constructs (WT-htt, mut-htt) were a kind gift of Prof. David Rubinsztein and the 4-repeat tau construct (4 R tau) was a kind gift of Dr. Romina Vuono. Cells were transfected using the GeneJuice Transfection Reagent (Millipore) following the manufacturer’s protocols.

### Osmolyte treatment

Cells were plated at 200 000 cells per well in 6-well plates and transfected the next day. 48 h post transfection, cells were treated with different concentrations of sucrose, NaCl, or mannitol for the indicated time. Varying amounts of concentrated osmolytes were added drop by drop directly onto the supernatant of cells to give a final osmolyte concentration in the extracellular medium after equilibration of 150 mM. After a 15-minute treatment (unless indicated otherwise), the supernatant was removed and the cells were scraped into 150 μl sample buffer to create a total cell lysate for analysis by SDS-PAGE and WB.

For the osmolyte treatment with triton study, the cells were plated and transfected as described above. 48 h post transfection, the cells were scraped from the dish, they were then spun down for 3 minutes at 800RPM, and resuspended in 100 μL medium. Cells were lysed using 1% triton with 1X Protease inhibitor cocktail (Roche). One drop of 5 M NaCl was added to the cell suspension to a final concentration of 150 mM. The cells were treated with NaCl for 15 minutes. After treatment, 50 μL of SB3x was added to the cell solution to create a total cell lysate for analysis by SDS-PAGE and WB.

### Heat, H_2_O_2_ and 6-OHDa treatment

Control experiments using heat, H_2_O_2_, or 6-OHDA treatments were performed on cells in suspension (as described above). Cells were treated with 1 mM, 10 mM or 100 mM of H_2_O_2_ or 5 μg/μL 6-OHDA (both from Sigma) for 15 minutes, or heated to 42 °C, 46 °C, 50 °C for 15 minutes. After the treatment, 50 μL of SB3x was added to the cell solution to create a total cell lysate for analysis by SDS-PAGE and WB.

### Analysis of cell survival

Cells were plated and transfected as described above. Before osmolyte treatment, the extracellular medium was changed to remove dead cells. Cells were treated with drops of 2.5 M sucrose to give a final concentration in the extracellular medium of 150 mM for 15 minutes. The medium was changed again and cells were incubated for the indicated periods of time (from 1 h to 6 h). For each time point, both the living cells attached to the bottom of the dish and the dead cells floating in the supernatant were collected. For the 0 h time point, samples were harvested immediately after treatment and the medium was not changed. The total cell lysate from living cells was created as described (in ‘Osmolyte treatment’). To collect dead cells, the supernatant was spun down in a bench top centrifuge at 18 000 × g for 5 minutes at 4 °C. The pellet was resuspended in 75 μL SB to create a total dead cell lysate for analysis by SDS-PAGE and WB.

### SDS and Urea resistance of aggregates

Cells were plated, transfected and treated as described above (in ‘Osmolyte treatment’). After treatment, the medium was removed and the cells were scraped into 200 μL RIPA (Thermo Scientific) or 200 μL RIPA + 7 M Urea (Sigma) with 1X Protease inhibitor cocktail. After 15 minutes, 50 μL of SB3x was added to the cells to create a total cell lysate for analysis by SDS-PAGE and WB.

### Osmolyte treatment on recombinant α-syn

50 μM recombinant α-syn was mixed with 40 μM ThT in 96-well plates. 5 M NaCl, 2.5 M sucrose and 2.5 M mannitol were added drop by drop to recombinant α-syn to give a final concentration of 150 mM. 0.1 μM and 0.25 μM seeds of preformed fibrils were used as positive controls. Seeds were produced by sonication for 10 s at 30% amplitude with a Bandelin Sonopuls HD 2070 sonication probe (Buch & Holm). Aggregation was monitored using ThT fluorescence.

### Thioflavin T (ThT) Fluorescence

96-well black Costar polystyrene microliter plates were used for these experiments. After sealing to prevent evaporation, the plate was placed in an Infinite M200 plate reader (Tecan Nordic AB). The plate was incubated at 37 °C, and the ThT fluorescence (excitation 450 nm, emission 482 nm) was measured every 5 min.

### FACS analysis

Post culture cells were spun down (350 g for 5 minutes) and washed with PBS x 2 after being transferred to 15 mL falcon tubes. They were then incubated with 1:500 of the viability stain (live dead zombie acqua, Biolegend 423101) for 30 minutes. The cells were then washed three times and resuspended in 2% PFA for 15 minutes. The PFA was washed off (x2) and the cells were resuspended in FACS buffer (Phosphate buffered saline, 0.1% Bovine serum albumin Merck Millipore 820451, 0.001% sodium azide, Sigma Aldrich S2002). Single colour flow cytometry was run using a BD LSR Fortessa. Cells were gated using Forward scatter A versus Forward scatter H to identify singlets and then gated on dead cells which were quantified as a percentage of singlets.

### Propidium Iodide (PI) staining

Supernatant was removed from the cells and placed into a new dish. Adherent cells dish were topped up with 500ul of fresh media. PI and DAPI were added to both plates (at a dilution of 1:20 and 1:5000, respectively). The plates were left for 20 mins at room temperature in darkness. Bright-light & fluorescent images of both sets of plates were taken at various time points (5, 15, & 60 minutes).

### Lactate dehydrogenase (LDH) assay

The Pierce LDH Cytotoxicity Assay Kit (88953) ThermoFisher was used, and the experiment was carried out as per the manufacturer’s instructions, although we adjusted the protocol for a 6 well plate assay. Briefly, 50 ul of supernatant from each 6 well plate was transferred into a 96 well plate in triplicate. 50 ul of reaction mixture was added to the supernatant and incubated for 30 mins at room temperature. The reaction was stopped by adding 50 ul of stop solution. Absorbance values were measured at 490 nm and 680 nm. To determine LDH activity, the 680 nm absorbance value was subtracted from the 490 nm absorbance, whilst taking into account the background signal from media/serum wells alone.

### MACS dead cell removal kit

The cells were collected, centrifuge at 300 g, and then the supernatant was removed completely. Cells were then resuspended in Dead Cell Removal MicroBeads, and mixed well. They were then incubated for 15 min at room temperature. The cell suspension was then placed in MACS Separator columns (prepared with Binding Buffer). After rinsing with Binding Buffer, the live cell fraction was collected. The dead cell fraction was forced out using the syringe plunger.

### SDS-PAGE and Western blot

Cells were lysed in lysis buffer (0.35 M TrisHCl pH 6.8, 2% sodium dodecyl sulfate (SDS), 5% β-mercaptoethanol (BME), Bromophenol blue, 60% glycerol, 3X). The total cell lysate was collected for analysis. Samples were sonicated twice for 5 seconds to fragment DNA and boiled for 5 minutes to denaturate proteins. For SDS-PAGE analysis, 15 μL of the total lysate was loaded onto NuPAGE Novex 10% Bis-Tris Protein gels (Invitrogen), run in MOPS buffer (invitrogen) at 100 V for the first 30 min and then run at 150 V, and visualised using Coomassie Brilliant Blue stain (45.4% methanol, 4.6% acetic acid, 0.1% Coomassie Brilliant Blue, 50% water). Background staining was removed using a distain buffer (5% methanol, 7.5% acetic acid, 87.5% water). For Western blot analysis, the proteins were separated as described for SDS-PAGE analysis. The proteins were then transferred to a PVDF membrane (Millipore) at 400 mA for 2 h at 4 °C in transfer buffer (6 g Tris base, 28.8 g glycine, 480 mL methanol, 1520 mL water). The membrane was blocked in 5% fat-free milk (Tesco) for 30 minute at room temperature (RT) and incubated with the following primary antibodies for 1 h at RT: α-syn (ab6162, Abcam, 1∶2′500 dilution), anti-GFP (ab290, Abcam, 1: 2′500 dilution), and anti-Tau (AT8, Thermo Scientific, 1:1′1000 dilution). The membrane was washed 3 times in TBS-Tween buffer (Thermo Scientific) for 15 min and incubated with the following secondary antibodies for 30 min at RT: Rabbit anti-Sheep IgG (H + L)-HRP Conjugate (BioRad Laboratories, 1:2′500 dilution), Goat anti-Mouse IgG (H + L)-HRP Conjugate (BioRad Laboratories, 1:10′000 dilution), Goat anti-Rabbit IgG (H + L)-HRP Conjugate (BioRad Laboratories, 1:10′000 dilution). The membrane was washed 3 times for 15 min. The protein bands were visualized using a SuperSignal West Pico Chemiluminescent Substrate (Thermo Scientific) in the dark room and the bands were exposed to X-ray films (GE Healthcare) in an exposure cassette for times ranging from 5 seconds to 5 minutes and the films were developed by conventional methods. The membranes were washed again and incubated overnight at 4 °C with an antibody to α-actin (AM4302, Thermo Fisher Scientific, 1∶5′000 dilution) following the same protocol. 0.5 μL of 10 μM recombinant α-syn fibrils was diluted in SB1x, sonicated and boiled for use as a positive control for aggregation on WB.

### Stereotaxic injections

C57/Black mice (from Charles River Labs, Margate, UK) were housed in group cages, with unrestricted access to food and water. The mice were anaesthetised with isoflurane, placed in a stereotaxic frame and were unilaterally injected in the striatum (co-ordinates: anterior–posterior: + 0.5; medial–lateral: + 0.21; dorsal–ventral: 3.0). A small hole was drilled in the exposed skull and a Hamilton syringe was used for the delivery of the AAV- α-syn (1 µL in total, at a rate of 0.5 µL/min). The mice were then sutured and allowed to recover in a heated recovery chamber. Eight weeks later, the mice were again anaesthetised with isoflurane, placed in a stereotaxic frame and unilaterally injected in the same striatum with 5 M NaCl (2 µL in total, at a rate of 0.5 µL/min). Control mice were injected with normal saline. The syringe was left in place for 5 min before being slowly removed. The mice were then maintained under isoflurane anaesthetisia until they were sacrificed (5, 10 or 30 minutes post injection; n = 3 at each time point) and their brains were removed for western blot analysis.

### *Ex vivo* analysis

Twenty week old Thy1-hSNCA (line 15) mice were sacrificed by cervical dislocation. Their brains were removed and washed in PBS, before being sectioned into coronal slices (approximately 500 µm thick) using a mouse brain slicer matrix (Zivic). The slices were immediately placed in 5 M NaCl for 1, 5 or 10 minutes. Efforts were made to keep the tissue fully immersed. Following this treatment, the slices were then removed and stored on dry ice for western blot analysis.

Brain tissue was homogenised using a glass homogeniser and extraction buffer (extraction buffer, Abcam ab193970-50 [20%], extraction buffer enhancer ab193971 [2%] and protease inhibitors [2%] diluted in ice cold tris-buffered saline). The homogenate was incubated on ice for 20 minutes and then centrifuged at 4 °C for 20 minutes at 14000 RPM. The supernatants were then removed and stored in 10 microliter aliquots. Protein concentration was determined using the Pierce BCA assay (ThermoScientific 23225) according to the manufacturers instructions.

Samples were adjusted to load a final weight of 10 µg of protein in 20 µl with LDS Sample Buffer (Invitrogen NuPage NP0007) and 0.1 M DTT (Invitrogen NuPage NP0009). They were then boiled at 70 °C to denature proteins. Once cooled they were loaded into NuPage 10% Bis-Tris gel with a sample ladder (Biorad 1610377). The gels were run for 30 mins at 100 V and then for a further 1.5 hours at 120 V with 1x MOPS running buffer (MOPS SDS running buffer NP0001, diluted from 20x). Protein was transferred from the gel to the membrane in ice cold transfer buffer (1x transfer buffer[Novex NuPage transfer buffer 20x NP0006], 20% methanol) running at 30 V for 3 hours.

The membrane was then blocked with 5% milk in TBST for 1 hour followed by an incubation with a mouse anti-human alpha synuclein antibody overnight (Abcam, ab1903; 1:2000). The antibody was washed off with TBST the following day (3 × 10 minute washes). The secondary antibody (goat anti mouse HRP conjugated) was incubated with the membranes in 5% blocking buffer for 1 hour at room temperature. The membranes were then washed 7 × 5 minutes in TBST on a shaker. The signal was then enhanced using the SuperSignal West Femto Kit (Thermo Scientific 34094) as per the manufacturers instructions. The signal was developed on a UVI TECH Alliance.

### Statistical analysis

Western blot bands were quantified by densitometry using ImageJ (https://imagej.nih.gov/ij/). At least 2 independent experiments were conducted for every Western blot analysis, with the results averaged. Statistical analysis was performed using GraphPad Prism software (version 6.0). All comparisons between groups of experiments were performed using ANOVA with Dunnett’s multiple comparisons test. All values are presented as means ± SEM, and statistical significance was set at P < 0.05 (*p < 0.05, **p < 0.005, ***p < 0.0005).

## Supplementary information

Supplemental Figures

Supplemental Information
