# Supplementary material for: Hyperosmotic stress induces cell-dependent aggregation of α-synuclein
Source: Sci Rep. 2019 Feb 19;9:2288. doi: 10.1038/s41598-018-38296-7 (PMC6381101; doi:10.1038/s41598-018-38296-7)

RESEARCH REPORT

Hyperosmotic stress induces cell-dependent aggregation of α-synuclein

Alexandra M C Fragniere, Simon R W Stott, Shaline V Fazal, Maria Andreasen, Kirsten Scott, & Roger A Barker

SUPPLEMENTAL FIGURES

SUPPLEMENTAL FIGURE 1.

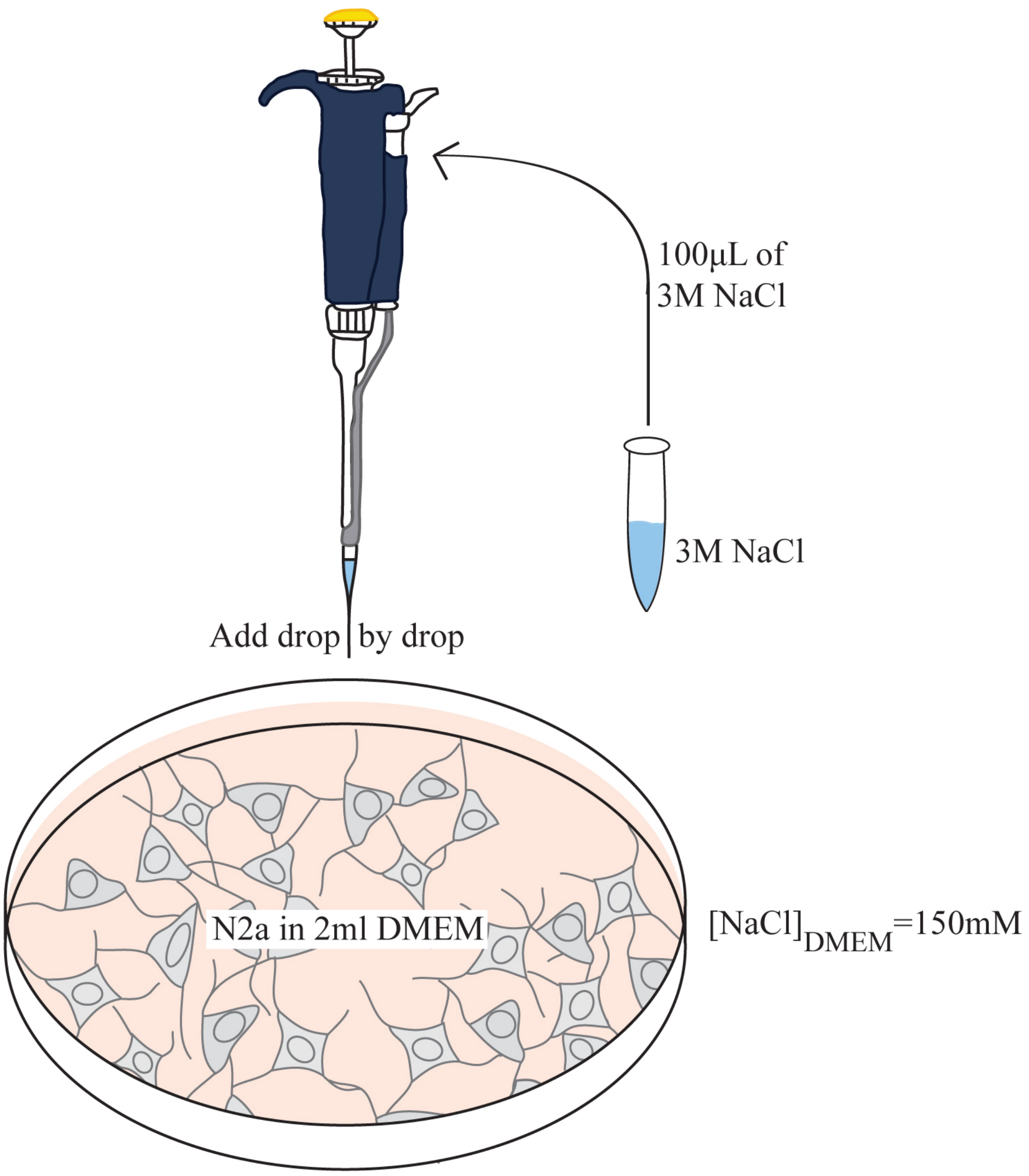

SUPPLEMENTAL FIGURE 2.

Hyperosmotic shock effect using HEK cells

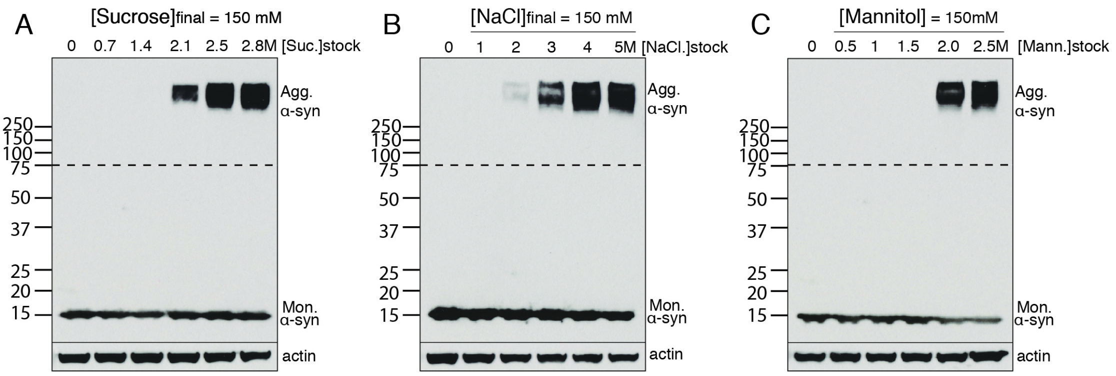

SUPPLEMENTAL FIGURE 3

The hyperosmotic shock induced aggregates are urea-resistant

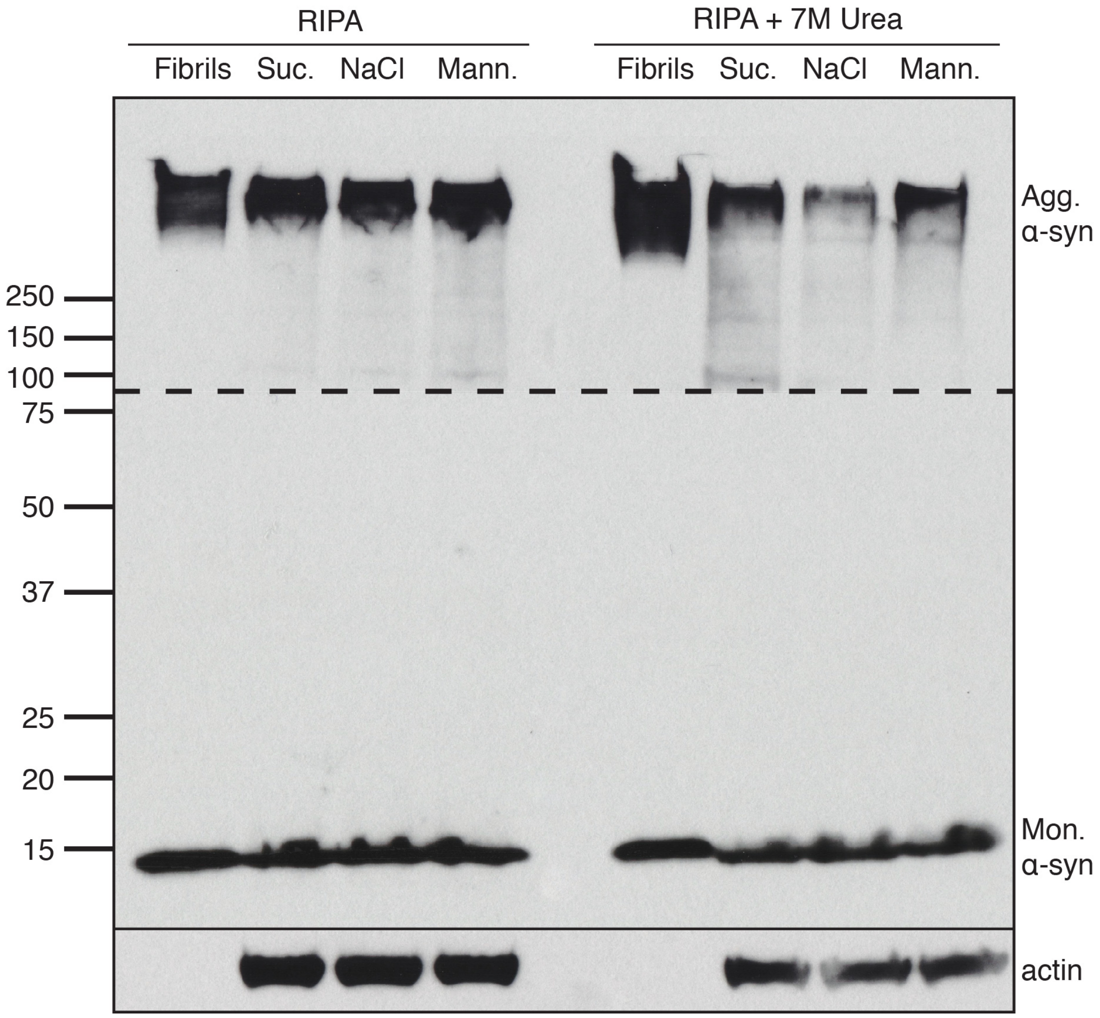

SUPPLEMENTAL FIGURE 4

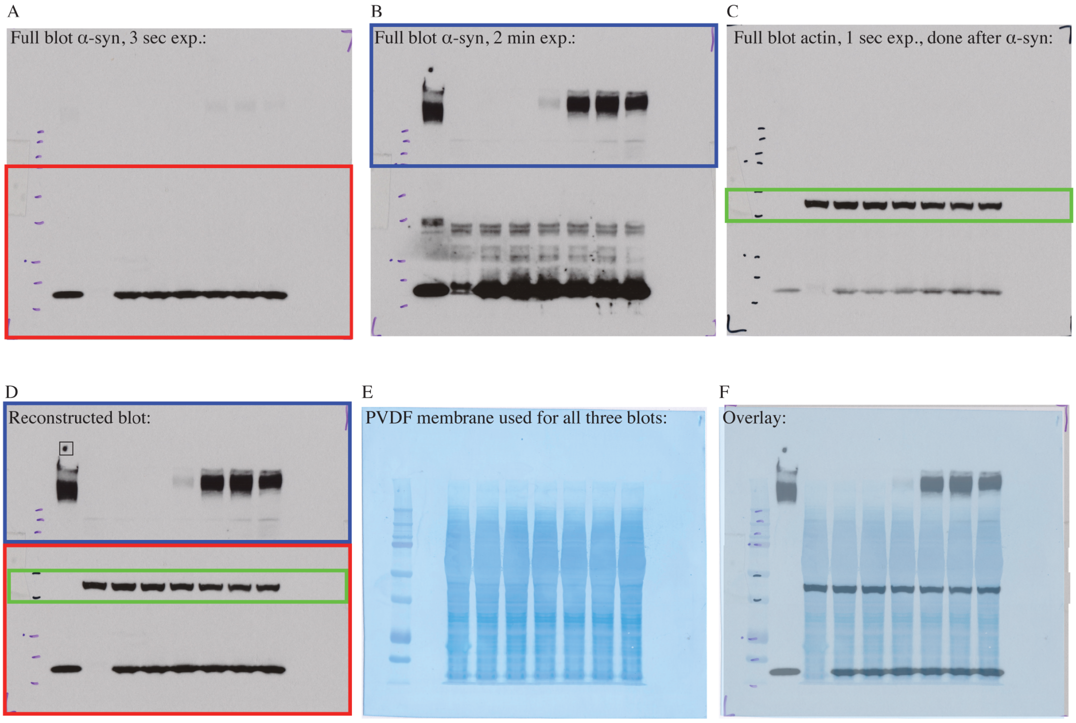

SUPPLEMENTAL FIGURE 5

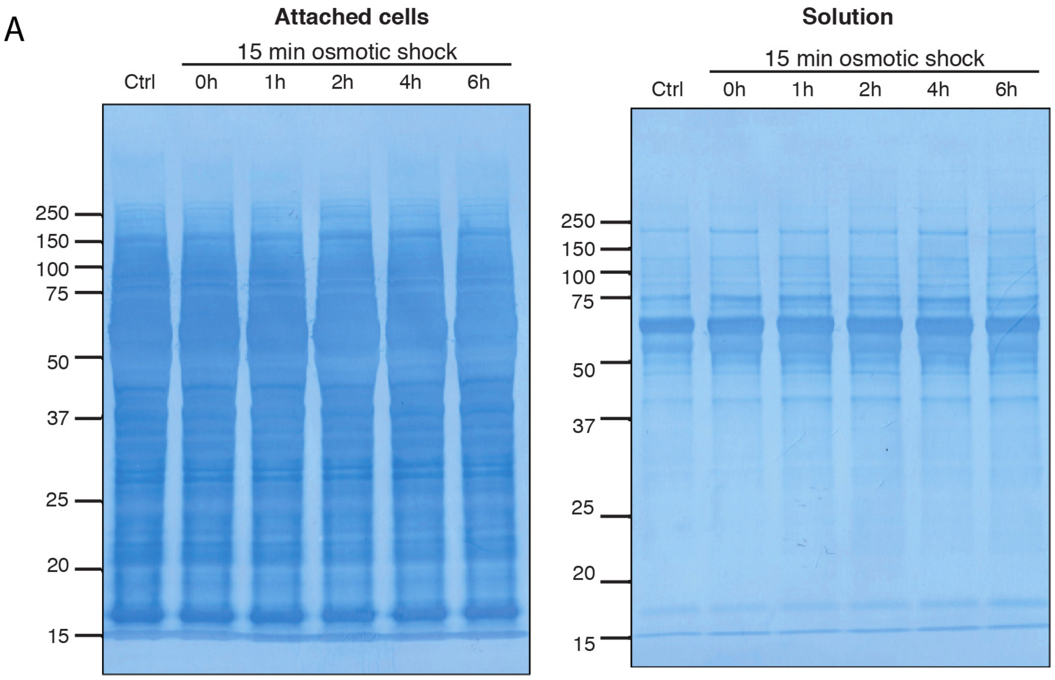

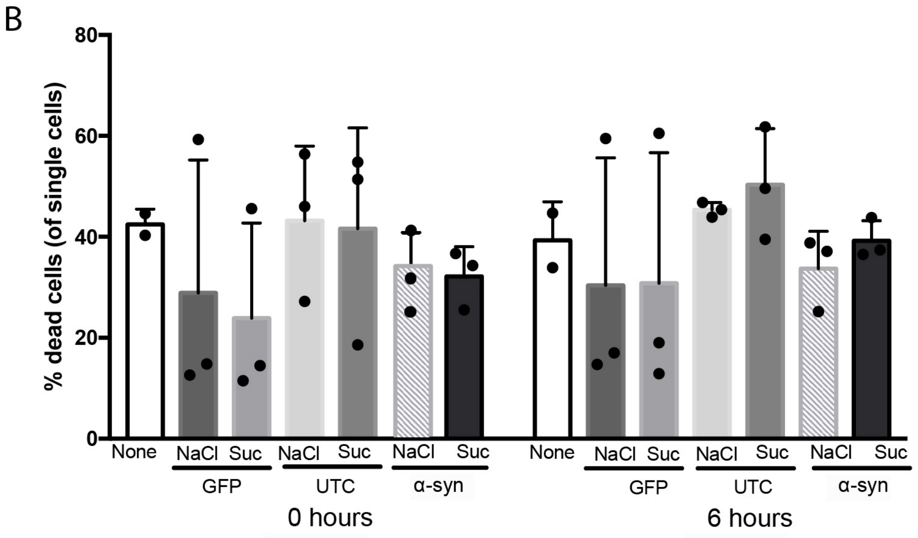

SUPPLEMENTAL FIGURE 6.

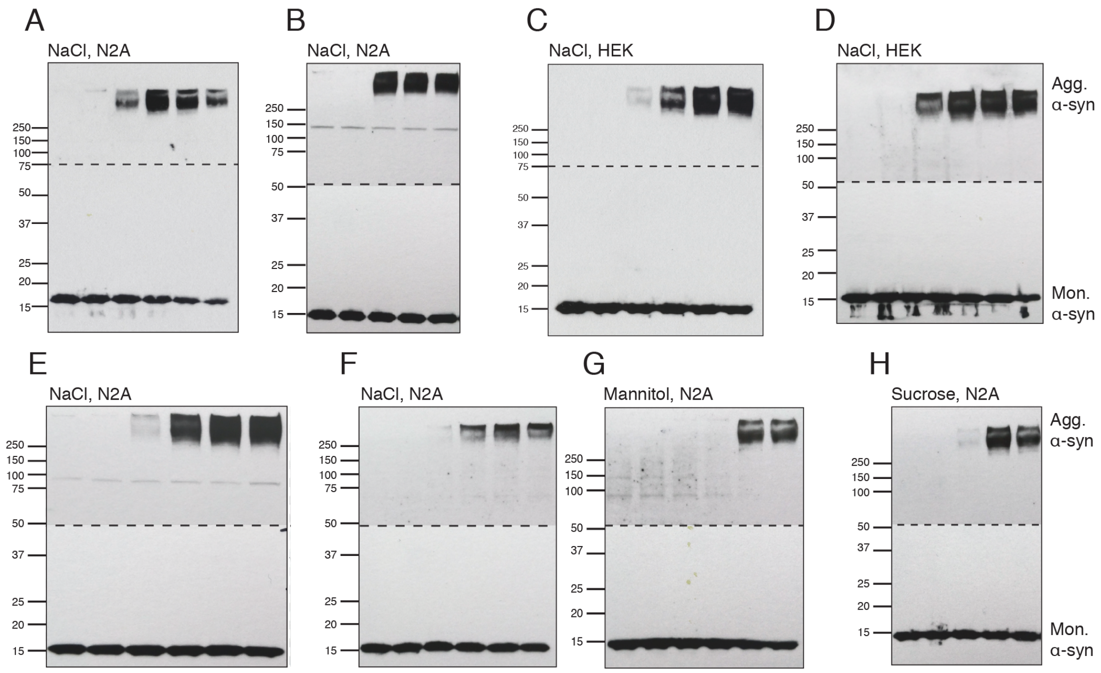

Supplement: Supplementary file 1 — Supplemental Figures [file 41598_2018_38296_MOESM1_ESM.pdf]
