# Supplementary material for: Hyperosmotic stress induces cell-dependent aggregation of α-synuclein
Source: Sci Rep. 2019 Feb 19;9:2288. doi: 10.1038/s41598-018-38296-7 (PMC6381101; doi:10.1038/s41598-018-38296-7)
Supplement: Supplementary file 2 — Supplemental Information [file 41598_2018_38296_MOESM2_ESM.docx]

RESEARCH REPORT

**Hyperosmotic stress induces cell-dependent aggregation of α-synuclein**

Alexandra M C Fragniere, Simon R W Stott, Shaline V Fazal, Maria Andreasen,

Kirsten Scott, & Roger A Barker

SUPPLEMENTAL INFORMATION

**Figure S1.** A schematic representation of 3M NaCl delivery.

**Figure S2.** Replication of the hyperosmotic shock effect on α-syn using HEK cells. The portion of the blots above the dashed lines was exposed for a longer time compared to the part of the blot below the dashed line.

**Figure S3.** The hyperosmotic shock induced aggregates are SDS-, urea-, and boiling resistant. After treatment with sucrose, NaCl or mannitol, cells were lysed in RIPA buffer (first half of the blot) or RIPA buffer with 7M urea (second half of the blot). After 15 minutes, SDS sample buffer was added and the samples were boiled for 10 min. α-syn fibrils were used as a positive control. The portion of the blots above the dashed lines was exposed for a longer time compared to the part of the blot below the dashed line.

**Figure S4.** Raw data of the WB shown in Figure 1A. (A) Full blot probed with α-syn antibody and exposed for 3 seconds. The red rectangle shows the portion of the gel used in the final figure. (B) Full blot probed with α-syn antibody and exposed for 2 minutes. The blue rectangle shows the portion of the gel used in the final figure. The additional lines observed above monomeric α-syn are background as they also appear in the untransfected cell sample loaded in the second well after the ladder. (C) Full blot probed with actin antibody and exposed for 1 second. The actin blot was performed the day after the α-syn blot. The green rectangle shows the portion of the gel used in the final figure. (D) Reconstructed blot using portions of the blots shown in A, B and C. The dot within the square was removed in the final figure. (E) PVDF membrane used for the three blots shown in A, B and C. In the first well after ladder, recombinant α-syn is not visible as only 5 pmoles were loaded. (F) Overlay of the PVDF membrane and the three blots.

**Figure S5.** (A) Coomassie staining of live cell lysate (attached cells) and dead cell lysate (Solution) collected at different time points following osmotic shock. (B) Graph displaying the results of the FACS analysis experiments (n=3) for NaCl and Sucrose (Suc) treated cells at 0 and 6 hours after osmotic shock. GTP and α-syn transfected cells were compared with untreated control (UTC) cells.

**Figure S6.** The induction of a-syn aggregation using hyperosmotic stress in cells is very robust and a highly reproducible result, which is not dependent on the osmolyte or the cell line used. The blots show 8 independent experiments performed using N2A or HEK cells treated with a range of osmolyte concentrations. A is part of figure 1 in the main manuscript, C is part of figure S2. They are presented here for comparison. The portion of the blots above the dashed lines was exposed for a longer time compared to the part of the blot below the dashed line.
